# Sodium Channel Myotonia Due to Novel Mutations in Domain I of Na_v_1.4

**DOI:** 10.3389/fneur.2020.00255

**Published:** 2020-04-29

**Authors:** Serena Pagliarani, Sabrina Lucchiari, Marina Scarlato, Elisa Redaelli, Anna Modoni, Francesca Magri, Barbara Fossati, Stefano C. Previtali, Valeria A. Sansone, Marzia Lecchi, Mauro Lo Monaco, Giovanni Meola, Giacomo P. Comi

**Affiliations:** ^1^Dino Ferrari Center, Department of Pathophysiology and Transplantation, University of Milan, Milan, Italy; ^2^Department of Neurology and INSPE, IRCCS Ospedale San Raffaele, Milan, Italy; ^3^Department of Biotechnology and Biosciences, University of Milano - Bicocca, Milan, Italy; ^4^Institute of Neurology, Area of Neuroscience, Fondazione Policlinico Universitario A. Gemelli, IRCCS, Rome, Italy; ^5^Fondazione IRCCS Ca' Granda Ospedale Maggiore Policlinico, Neurology Unit, Milan, Italy; ^6^Department of Neurorehabilitation Sciences, casa di Cura del Policlinico, Milan, Italy; ^7^Neurorehabilitation Unit, University of Milan; The NEMO (NEuroMuscular Omniservice) Clinical Center, Milan, Italy; ^8^Department of Biotechnology and Biosciences and Milan Center for Neuroscience, University of Milano - Bicocca, Milan, Italy; ^9^MIA (Myotonics in Association), Portici, Italy; ^10^Fondazione IRCCS Ca' Granda Ospedale Maggiore Policlinico, Neuromuscular and Rare Diseases Unit, Milan, Italy

**Keywords:** myotonia, sodium channel myotonia, founder effect, channelopathy, Na_v_ 1.4, mexiletine

## Abstract

Sodium channel myotonia is a form of muscle channelopathy due to mutations that affect the Na_v_1.4 channel. We describe seven families with a series of symptoms ranging from asymptomatic to clearly myotonic signs that have in common two novel mutations, p.Ile215Thr and p.Gly241Val, in the first domain of the Na_v_1.4 channel. The families described have been clinically and genetically evaluated. p.Ile215Thr and p.Gly241Val lie, respectively, on extracellular and intracellular loops of the first domain of the Na_v_1.4 channel. We assessed that the p.Ile215Thr mutation can be related to a founder effect in people from Southern Italy. Electrophysiological evaluation of the channel function showed that the voltage dependence of the activation for both the mutant channels was significantly shifted toward hyperpolarized potentials (Ile215Thr: −28.6 ± 1.5 mV and Gly241Val: −30.2 ± 1.3 mV vs. WT: −18.5 ± 1.3 mV). The slow inactivation was also significantly affected, whereas fast inactivation showed a different behavior in the two mutants. We characterized two novel mutations of the *SCN4A* gene expanding the knowledge about genetics of mild forms of myotonia, and we present, to our knowledge, the first homozygous patient with sodium channel myotonia.

## Introduction

Myotonia is an impaired muscle relaxation after a voluntary muscle contraction and is the main feature of a group of heterogeneous skeletal muscle channelopathies named non-dystrophic myotonias (NDMs). NDMs are caused by mutations in *CLCN1* and *SCN4A* genes, coding, respectively, for the chloride (ClC-1) and sodium (Na_v_1.4) muscle channels (1).

Na_v_1.4, the α-subunit of the sodium channel complex, mainly expressed in skeletal muscle, is formed by 1836 amino acids and displays a tetrameric structure composed of 4 domains (DI-DIV), each including six transmembrane α-helices (S1–S6). The inner part of the channel contains a pore (S5–S6 from each domain) where sodium ions flow through thanks to four voltage sensors (S1–S4 from each domain). After an action potential, the cell membrane enters a “refractory period” when it becomes inexcitable thanks to a double mechanism of Na_v_1.4 inactivation, either related to a fast or a slow kinetic. Depending on their nature and localization, mutations in the *SCN4A* gene may enhance or decrease muscle excitability, and elicit different physiological reactions by the mutated channel which are related to different muscle diseases. These now include autosomal dominant sodium channel myotonia (SCM), paramyotonia congenita, hyperkalemic periodic paralysis, hypokalemic periodic paralysis, congenital recessive myasthenia, and the autosomal recessive congenital myopathy with hypotonia (2) and sudden infant death syndrome [SIDS; (3)].

SCMs are characterized by the absence of weakness and often by cold sensitivity and muscle pain (4). Clinical phenotype is highly variable, ranging from a severe neonatal presentation, which recently has been associated to SNEL (severe neonatal episodic laryngospasm) and stridor (5), passing through classical SCM with onset in the first or second decade, to mild, late-onset phenotypes (6).

To date, over 70 mutations inherited in autosomal dominant fashion have been reported in the *SCN4A* gene and related to SCM and periodic paralyzes. Some of these mutations are found in the first domain of the sodium channel and have been described to cause both SCM and paramyotonia congenita (7–11). Most of the mutant channels have been extensively investigated *in vitro*, indicating enhanced activation and/or impaired fast inactivation as the major mechanisms underlying the myotonia phenotype (1).

Here, we present the characterization of the novel mutations p.Ile215Thr and p.Gly241Val in the first domain of Na_v_1.4. Ile215Thr is shared by six unrelated families from Southern Italy. Of note, one of the affected individuals is homozygous for this mutation but he does not share any of the features recently described for autosomal recessive congenital myopathy (12). Data from electrophysiological assays positively correlated with pathogenicity for both variants.

## Materials and Methods

### Patients

**Figure 1:**
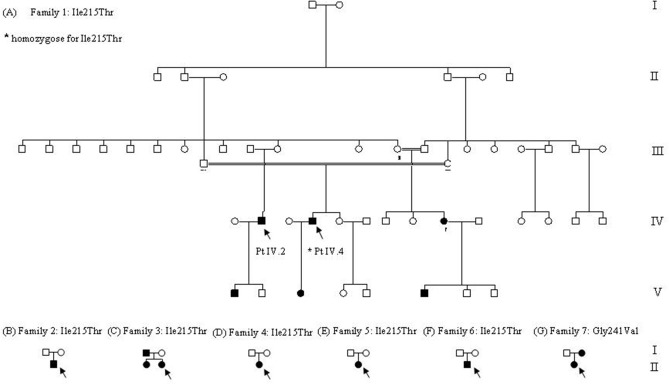
Family trees. **(A–F)** Family trees of families that carried p.Ile215Thr. In the picture, we indicated by arrows the two probands, IV.2 heterozygote, and IV.4 homozygote for the target mutation. As depicted by the doubled lines, two marriages between relatives occurred in this family, and only one homozygote was generated (*). **(G)** Family tree of the only family carrying p.Gly241Val.

This study was carried out in accordance with the recommendations of Fondazione IRCCS Ca' Granda Ospedale Maggiore Policlinico of Milan. All subjects gave written informed consent for genetic analysis in accordance with the Declaration of Helsinki. In families 2 and 4, we could analyze only the proband, while for family 5, we could analyze the proband and her father: the mother probably had the same symptomatology of her daughter but was not available for genetic testing. In family 6, we could analyze the proband and his mother (Figure 1).

### Genetic Analysis

Genomic DNA was extracted from blood samples using FlexiGene DNA Kit (Qiagen, Hilden, Germany). DM1 and DM2 expansion and CLCN1 mutations were excluded in the probands of each family. PCR fragments containing all the 24 coding exons and intron–exon junctions of *SCN4A* were amplified (primer sequences and conditions are available upon request). The fragments were directly sequenced using the same PCR primers and Big Dye Terminator Cycle Sequencing Kit in an automated sequencer 3130 (Applied Biosystems, Foster City, USA). Sequences were aligned using SeqScape software (Applied Biosystems) and compared to sequences NG_011699 and NM_000334 from NCBI. To confirm the results obtained, amplification and sequencing were repeated and the novel variant was checked in 200 Italian controls.

Microsatellite markers analysis was performed in order to verify the hypothesis of a founder effect for the c.644T>C mutation. Families 1, 2, and 3 were genotyped using markers D17S787, D17S944, D17S949, and D17S785 from ABI PRISM Linkage Mapping Set v2.5 (panels 23 and 24; Applied Biosystems) and markers D17S1792, D17S113, D17S584, D17S789, and D17S1786 annotated in NCBI (amplification conditions are available upon request). The fragments were resolved in an automated sequencer 3130 and the results were analyzed with GeneMapper Software 5 (Applied Biosystems).

### Cloning of Na_v_1.4 and Electrophysiological Characterization in tsA Cells

Total RNA from a pulled skeletal muscle specimen (Human Total RNA Master Panel II, Clontech, Mountain View, USA) was reverse-transcribed with Ready-To-Go kit (GE Healthcare, Little Chalfont, UK). *SCN4A* cDNA was amplified with specific primers (primer sequences and conditions are available upon request) and cloned in mcs1 of pVITRO2-mcs vector (Invivogen, San Diego, USA); GFP cDNA was cloned in mcs2. Clones were checked by sequencing. Site-directed mutagenesis was performed using the Quick-change II kit (Stratagene, Santa Clara, USA) and specific primers for the mutation of interest. The mutant clones were verified again by direct sequencing.

The functional characterization of mutant channels was performed by transiently transfecting vectors containing mutated human cDNA in tsA human kidney epithelial cells, derived from HEK 293 cell line. The evoked sodium currents were recorded by the patch-clamp technique in the whole-cell configuration. Current recordings were performed 48–72 h after the transfection, using the MultiClamp 700A amplifier and pClamp 8.2 software (Axon Instruments, USA) for data acquisition. Pipette resistance was about 1.5–1.8 MΩ; capacitance and series resistance errors were compensated (85–90%) before each protocol run to reduce voltage errors to <5% of the protocol pulse. Recordings were performed at room temperature. During the experiments, cells were maintained in a physiological extracellular solution, containing (mM): NaCl 130, KCl 5, CaCl_2_ 2, MgCl_2_ 2, glucose 5, and Hepes 10. The solution for the patch electrodes was composed by (mM): CsF 105, CsCl 27, NaCl 5, MgCl_2_ 2, Hepes 10, and EGTA 10.

**Figure 2:**
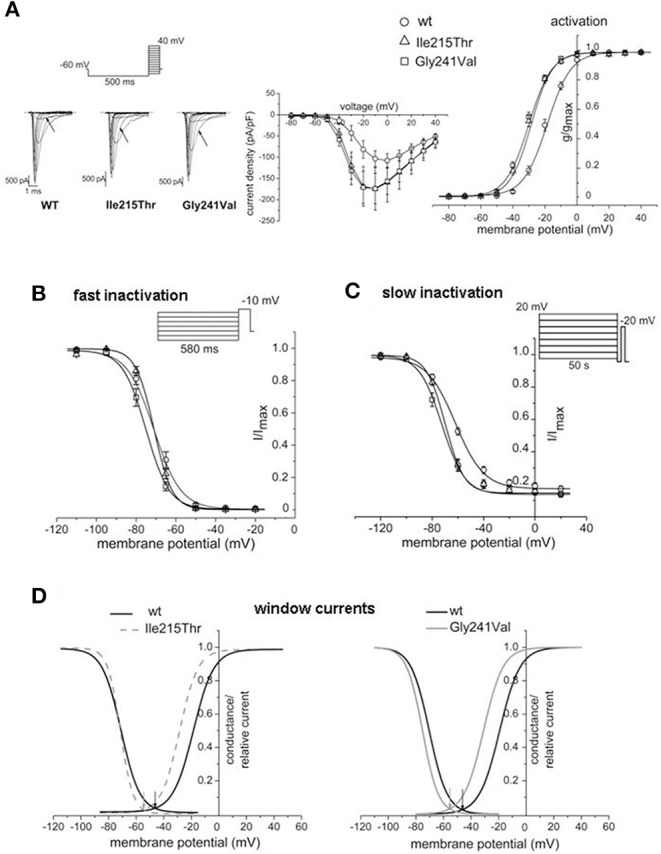
Effects of p.Ile215Thr and p.Gly241Val mutations on Na_v_1.4 functional properties. **(A)** Representative traces of whole-cell sodium currents; the arrows indicate the currents in response to the voltage step at −30 mV. Normalized current/voltage relationship was obtained by normalizing current by cell capacitance. Voltage dependence of activation was studied by applying the protocol represented and by fitting the normalized conductance by a Boltzmann curve. Comparison with WT indicates a shift of −10 and −12 mV for the curves of p.Ile215Thr and p.Gly241Val, respectively. **(B)** Voltage dependence of fast inactivation. A mild shift toward hyperpolarized potentials is observed only for p.Gly241Val. **(C)** Slow inactivation properties studied with long conditioning voltage pulses. Changes in the *V*_1/2_ are observed for both p.Ile215Thr and p.Gly241Val. **(D)** Window currents obtained overlapping activation and inactivation curves; they identify the voltage range in which Na_v_1.4 channels are available for opening. p.Ile215Thr and p.Gly241Val show to be available to open in a more hyperpolarized voltage range compared to WT.

Voltage dependence of activation was determined by the protocol represented in Figure 2A. Starting from a holding potential of −60 mV, cells were conditioned at −100 mV for 500 ms and successively tested by depolarizing potentials in 10-mV increments, from −80 to +40 mV. The properties of *fast* inactivation were studied using a double pulse protocol composed of 580-ms conditioning steps of varying voltages, from −110 to −20 mV, followed by a constant test pulse to −10 mV (Figure 2B). *Slow* inactivation was investigated by applying a conditioning 50-s pulse of increasing voltage (from −120 to 20 mV) to induce slow inactivation, followed by a 100-ms step to −130 mV for fast inactivation recovery and a test pulse to −20 mV (Figure 2C). Data were fitted with Boltzmann functions, *y* = 1/(1 + exp[(*V*–*V*_1/2_)/*k*]) or *y* = *I*_o_ + ([1–*I*_o_]/[1 + exp((*V*–*V*_1/2_)/*k*)]) for the slow inactivation, where *V* is the membrane potential, *V*_1/2_ is the half-maximal activation or inactivation voltage, *k* is the slope factor, and *I*_o_ is the non-zero current level.

All data are presented as mean ± SEM. Statistical evaluation was performed using one-way analysis of variance (ANOVA) with statistical significance set at *p* < 0.05.

## Results

### Genetic Analysis

Direct sequencing of the *SCN4A* gene of the proband of each family revealed two novel variants in the first domain of Na_v_1.4, not detected among healthy family members and among 200 Italian controls.

Patients from six unrelated families (families 1–6) shared the same variant in exon 5, c.644T>C, that causes an amino acid change at position 215 from isoleucine into threonine (p.Ile215Thr). Ile215 is located within the extracellular loop S3–S4 of the first domain (DI S3–S4 linker). Patient IV.4 from family 1 was born from consanguineous parents and carries the p.Ile215Thr mutation in homozygosis. Genetic analysis of family 7 revealed a c.722G>T variant in exon 6 leading to the amino acid change p.Gly241Val. Gly241 is located in the cytoplasmic loop S4–S5 (DI S4–S5 linker). Both amino acids, Gly241 and Ile215, are highly conserved through orthologs. Bioinformatic analysis of the variants was performed using free online tools. Polyphen 2, SIFT, and Mutation Taster predicted that both variants could affect protein function, while PMUT predicted a neutral effect. Both variants are not described either in gnomAD or in 1,000 G.

### Clinical Examination of Patients Carrying p.Ile215Thr

The examined families showed a spectrum of clinical manifestations, ranging from asymptomatic, and subclinical subjects to patients with clear myotonic signs (Table 1). All the patients carrying p.Ile215Thr showed electrical myotonia at EMG examination.

**Table 1:** Clinical signs and instrumental data.

	**lle215Thr**													**Gly241Val**	
**Family**	**Family 1**						**Family 2**	**Family 3**			**Family 4**	**Family 5**	**Family 6**	**Family 7**	
**Patients**	**Patient IV.2**	**Patient V.1**	**Patient IV.9**	**Patient V.6**	**Patient IV.4**	**Patient V.3**	**Patient II.1**	**Patient II.2**	**Patient I.1**	**Patient II.1**	**Patient II.1**	**Patient II.1**	**Patient II.1**	**Patient II.1**	**Patient I2**
Age at last examination (years)	59	28	63	26	67	32		35	65	32	48	28	32	36	80
Onset	Subclinical	Asymptomatic	50 years		Childhood	32 years	35 years	Childhood	Asymptomatic	Asymptomatic	45 years	Childhood	Adolescence	early adulthood	adulthood
Clinical myotonia	Mild eye-lid myotonia	Percussion myotonia	Very mild grip myotonia	Very mild grip myotonia	Paramyotonia in the orbicularis oculi and grip myotonia	No myotonia	Fluctuating grip and percussion myotonia	Percussion myotonia	Lid-lag	Mild grip myotonia	Tongue	No	No	lid-lag, grip myotonia, spontaneous and percussion myotonia of the lower limbs	
Triggers for myotonia	Absent	Absent	Cold temperature	Cold temperature	Exercise	Pregnancy	Cold temperature	Cold temperature			Cold temperature	Fasting, exercise, rest, stress		cold temperature	
Paradoxical myotonia	Absent	Absent	Absent	Absent	Yes	No					No	Yes			
Stiffness	Absent	Absent	In the morning	In the morning	Yes	No	Yes	Severe			After exsercise at hands	Yes		lower limbs	mild
EMG	Myotonic discharges	Myotonic discharges	Myotonic discharges	Myotonic discharges	Myotonic discharges	No	Myotonic discharges	Myotonic discharges	Myotonic discharges	na	Myotonic discharges	Myotonic discharges	Myotonic discharges	myotonic discharges	na
Warm-up					No	No					No	No			
Muscle pain			Yes	Yes	Yes	No	Yes	Yes			Yes	Yes	No	yes, lower limbs	
Muscle weakness					No	No	no				Progressive and diffuse weakness	No			
Contractures			Muscle cramps	Muscle cramps	No	No					No		Contractures + muscle cramps		
Hypertrophy	Yes	Diffuse			No	No		Diffuse			no		Hypertrophy of calves	mild	
CK level (U/L)	300–500	223	na	200	300	Normal	Normal	na	Normal	Normal	na	Normal	Normal	normal	normal
Drugs					Mexiletine	No	No	Mexiletine and clonazepam				Phenytoin, quinine, mexiletine	No	mexiletine, acetazolamide, carbamazepine, phenytoin	
Response to drugs					Good response to mexiletine—Worsening of myotonia after statin therapy			Improvement in the frequency, duration and severity of the episodes of stiffness				Phenytoin: not effective quinine: effective, but not tolerated mexiletine: good response		All drugs were not effective	
Biopsy	Mild myopathic abnormalities				Increased fiber size variation, type I fibers atrophy, internal nuclei			Mild myopathic abnormalities - type 2B deficiency				Normal. Increased acid fosfatase activity.	Normal		
Other diseases			Mild ptosis ogival cleft		Diabetes, hypercholesterolemia and hypertrigliceridia	Mutation of gene of MTHFR		Hypothyroidism			Low serum vitamin D				

*In grey, the family that does not share the p.Ile215Thr*.

Family 1 is a large Italian family originally from Sicily that could be traced through five generations, the last two showing family members with similar symptoms (Figure 1A). The first proband (Patient IV.2) was a 60-year-old man with no clinical symptoms and no muscle weakness that had just an incidental finding of high CK values (300–500 UI/l). At neurological examination, muscle hypertrophy, and mild eyelid myotonia were noticed without percussion myotonic phenomenon in other muscles. EMG showed high-frequency repetitive discharges without myopathic changes and muscle biopsy revealed mild myopathic abnormalities. Cold sensitivity was not reported. His elder son, who, at the first examination, was 23 years old, has a very similar phenotype with diffuse muscle hypertrophy, percussion myotonia, and a mild CK increase (223 U/L). One cousin (Patient IV.4, the second proband of this family) underwent neurological examination in another hospital when he was 60 years old for mild CK elevation (300 U/L) and stiffness occurred after statin administration and persisting despite therapy discontinuation. He was born from parents that were first cousins, without reported symptoms, and he was homozygote for p.Ile215Thr. Past medical history was positive for diabetes treated with oral antidiabetic drugs. Since childhood, he complained of mild difficulties in starting leg movements that did not interfere with activity of daily living. Neurological examination showed paramyotonia in the orbicularis oculi and grip myotonia. Muscle mass and strength were normal. Laboratory studies demonstrated normal thyroid hormone levels. EMG showed myotonic discharges in all the examined muscles. Muscle biopsy showed mild non-specific abnormalities such as increased fiber size variation, internal nuclei, and type 1 fiber atrophy. He was treated with mexiletine and he was a good responder. His 37-year-old daughter, which is an obligate heterozygous carrier for p.Ile215Thr, was asymptomatic, and during neurological examination, no clinical myotonia was noticed. She complained of a generalized stiffness only during pregnancy. The CK values were normal.

Patient II.2 from family 2 sought neurological attention at 35 years of age because of diffuse muscle pain and stiffness with symptoms worsening with cold exposure (Figure 1B). He had never experienced weakness or episodic paralysis. Neurological examination was normal except for very mild and fluctuating grip and percussion myotonia. CK was normal. Family history was negative for neuromuscular diseases. His family came from Sicily.

In family 3, which originates from Sicily, the proband is a 29-year-old woman (Patient II.2) who was seen because of muscle pain, worsening with cold exposure (Figure 1C). Symptoms had started in childhood with delayed relaxation of hand and leg muscles. She was also bothered by diffuse muscle hypertrophy despite the fact that she was not doing any intense physical activity. During the last decade, she experienced worsening of the symptoms with additional short episodes of lower limb episodic paralysis. Muscle biopsy showed mild unspecific features with type 2B fiber deficiency. Hypothyroidism was detected in the last 3 years. She was placed on replacement therapy and mexiletine 200 mg bid with improvement in the frequency, duration, and severity of the episodes of stiffness. Her 66-year-old father was asymptomatic and neurologic exam was normal except for lid-lag. CK were normal. Myotonic discharges were found on EMG. Her 33-year-old sister complained of mild grip myotonia. Neurologic exam was normal. CK were normal. She refused EMG studies.

In family 4, the proband is a 48-year-old woman (Patient II.1) who complained of a very mild myotonia that was aggravated by cold and stiffness at hands after exercise (Figure 1D). Clinical examination revealed only a tongue myotonia. She also complained of a diffuse muscle weakness but the strength was normal during manual testing. EMG showed myotonic discharges in all examined muscles. Her family came from Sicily.

Patient II.1 from family 5 underwent medical examination when she was 28 years old for muscle pain that started in childhood (Figure 1E). Symptoms worsened with fasting, rest, exercise, and emotional stress. Neurological examination did not reveal clinical myotonia. EMG showed myotonic discharges without myopathic changes and muscle biopsy was normal. She had a good response to mexiletine treatment while phenytoin was not effective and quinine was effective but was not tolerated. Her mother probably had the same symptomatology but was not available either for clinical or for genetic examination. Her father was negative at genetic testing.

Patient II.1 from family 6 complained of contractures and muscle cramps not related to exercise or cold temperature from the age of 20 (Figure 1F). Clinical myotonia was not detected but EMG showed myotonic discharges. Muscle biopsy was normal. At the last examination when he was 32 years old, he complained of significant fatigue that let him work for only a few hours a day. His family came from Calabria.

### Clinical Examination of Patients Carrying p.Gly241Val

This 36-year-old woman (family 7, patient II.1; Figure 1G) came to neurological attention because of muscle pain and stiffness predominantly in the lower limbs, which worsened with cold exposure. Symptoms had been present since her early adulthood. She never reported episodes of muscle weakness. Neurological examination was normal except for muscles of mildly increased bulk and myotonia: lid-lag, grip myotonia with warm-up phenomenon, and spontaneous and percussion myotonia in the proximal lower limb muscles were clearly evident. Treatment with the common anti-myotonic drugs (carbamazepine, phenytoin, acetazolamide, and mexiletine) was ineffective. There were, however, no major functional limitations in everyday activities. CK was normal. Needle EMG showed abundant myotonic discharges especially in proximal lower limb muscles. Her 80-year-old mother complained of mild muscle stiffness since adulthood (Patient I.2). She had never sought medical attention because she had never experienced functional limitations. CK was normal. EMG was not available.

### Founder Effect

For five of the six independent Italian families that carried the p.Ile215Thr, we were able to verify the origin from Southern Italy; in particular, four families came from Sicily and one family came from Calabria. Therefore, we hypothesized that a founder effect could be responsible for the distribution of this mutation (Table 2). Microsatellite marker analysis in these families revealed that affected members from families 1 and 3 share the same allele spanning from 61.4 to 68.5 Mb in chromosome 17. Patient II.1 from family 2 share a smaller region of about 2,5 Mb with the other two families.

**Table 2:** Microsatellite analysis showing haplotypes for five families carrying the p.Ile215Thr.

**Marker name**	**D17S787**	**D17S944**	**SCN4A**	**D17S1792**	**D17S113**	**D17S584**	**D17S789**	**D17S1786**	**D17S949**	**D17S785**
Physical position (Mb)	53.28	61.4	62	63.06	63.9	65.12	66.63	67.5	68.5	75
		c.644 T>**C**								
Family 1 Patient IV.2	142	325	**C**	302	177	161	185	181	215	175
Family 1 Patient V.1	142	325	**C**	302	177	161	185	181	215	175
Family 1 Patient IV.9	142	325	**C**	302	177	161	185	181	215	175
Family 1 Patient V.6	142	325	**C**	302	177	161	185	181	215	175
Family 1 Patient IV.4	142	325	**C**	302	177	161	185	181	215	175
	142	325	**C**	302	177	153	183	177	221	173
Family 2 Patient II.1	144	325	**C**	302	177	161	185	181	215	169
Family 3 Patient II.2	154	323	**C**	302	179	161	187	177	217	169
Family 3 Patient II.1	154	323	**C**	302	179	161	187	177	217	169
Family 3 Patient I.1	154	323	**C**	302	179	161	187	177	217	169

*In grey, markers in the minimal identity region. In bold, the allele in linkage on the disease gene*.

### Functional Studies

**Figure 3:**
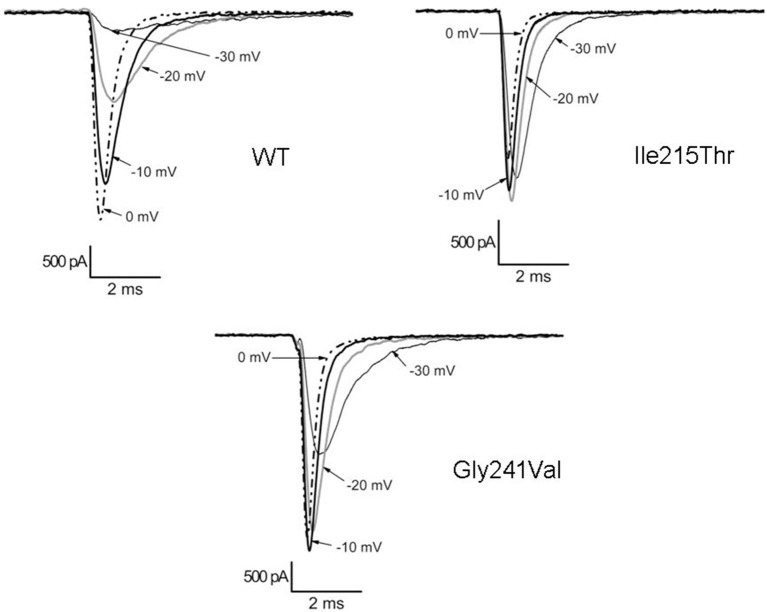
Traces illustrating the current decay for WT and mutant channels.

**Figure 4:**
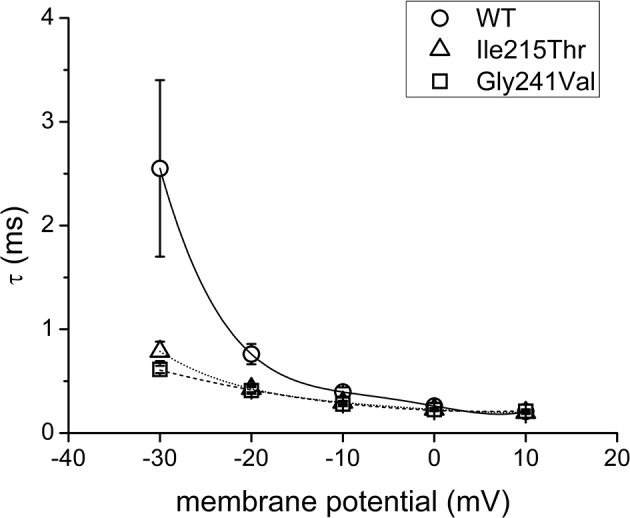
Curves illustrating the time constants (τ) for WT and mutant channel current decay in the range of potentials from −30 to 10 mV.

Ile215Thr and Gly241Val mutants expressed in tsA cells were able to generate functional channels as demonstrated by the sodium currents they evoked. Measured current densities were 176 ± 50 and 172 ± 34 pA/pF, respectively, for Ile215Thr (*n* = 10) and Gly241Val (*n* = 13) and were not significantly different from that of WT currents (113 ± 11 pA/pF, *n* = 13). Analysis of channel properties was performed by the voltage protocols described in Methods and represented in Figure 2. The voltage dependence of the activation for both the mutants was significantly shifted toward hyperpolarized potentials; the corresponding values of *V*_1/2_ were, respectively, −28.6 ± 1.5 mV for Ile215Thr and −30.2 ± 1.3 mV for p.Gly241Val vs. −18.5 ± 1.3 mV for WT channels (Figure 2A). The slow inactivation was also significantly affected; *V*_1/2_ was translated of about −7.5 mV for p.Ile215Thr and of −10 mV for p.Gly241Val (Figure 2C). Moreover, for p.Ile215Thr, the slope of the voltage dependence curve was also significantly changed (Table 3). Concerning the voltage dependence of fast inactivation, the two mutants manifested different behavior: no effects were recorded for p.Ile215Thr, whereas a modest but significant shift was evident for p.Gly241Val (Figure 2B; *V*_1/2_ and *k* values listed in Table 3). By fitting the decay of the individual current traces (obtained by protocol in Figure 2A) using a monoexponential curve from 90% of peak (Figure 3), the time constants (τ) of inactivation onset at −20 mV for both mutants were significantly different from WT, and shifted ~10 mV left (Figure 4; Ile215Thr vs. WT, *p* = 0.01; Gly241Val vs. WT, *p* = 0.009, ANOVA test, *n* = 5 cells for each condition).

**Table 3:** Voltage dependence of activation, fast inactivation, and slow inactivation.

	**Activation**		**Fast inactivation**		**Slow inactivation**	
	***V*_**1/2**_ (mV)**	***k* (mV)**	***V*_**1/2**_ (mV)**	***k* (mV)**	***V*_**1/2**_ (mV)**	***k* (mV)**
WT	−18.5 ± 1.3	7.4 ± 0.4	−71.2 ± 1.3	6.8 ± 0.5	−62.2 ± 1.1	11.3 ± 0.8
Ile215Thr	−28.6 ± 1.5^***^	6.6 ± 0.5	−71.0 ± 1.1	4.6 ± 0.4	−69.7 ± 0.9^**^	7.3 ± 0.4^**^
Gly241Val	−30.2 ± 1.3^***^	7.2 ± 0.5	−75.4 ± 1.4^*^	5.9 ± 0.5	−72.4 ± 1.1^***^	8.9 ± 0.5

Values are expressed as means ± SEM. Asterisks represent significant differences between mutants and WT channels:

* p < 0.05,

** p < 0.01,

*** *p < 0.001*.

## Discussion

In the present work, two additional novel dominant mutations, both sited in the first domain of the Na_v_1.4 α-subunit, were extensively studied. The p.Ile215Thr and p.Gly241Val are, respectively, located within the S3–S4 extracellular linker and the S4–S5 intracellular loop. We consider both variants to be pathogenic because (i) these mutations segregate with affected family members and are absent in 400 Italian chromosomes; (ii) they involve highly conserved amino acids; and (iii) functional analysis show altered behaviors compared with wild-type channel.

Both the mutations caused two principal effects on channel behavior: an increase in the open probability and in the slow inactivation. The first one concurs in the enhancement of channel availability during depolarization and resulted in a shift of the window current of respectively −9 and −10 mV for Ile215Thr and Gly241Val (Figure 2D). The second effect could prevent prolonged depolarization of the membrane, which is related to paralysis and episodes of weakness, as previously described by Petitprez et al. (10). These behaviors represent common traits of myotonic mutants and they have been shown for other mutations in sodium channel domain I (10). The alterations we observed in the activation properties are coherent with hyperexcitability phenomena and the myotonic symptoms referred by the patients. However, in both Ile215Thr and Gly241Val mutants, the shift in the voltage dependence of activation toward hyperpolarized potentials also accelerated the rate of the open-state inactivation, a mechanism consistent with a loss-of-function effect. This characteristic has already been observed by Petitprez et al. (10), but has not been described for other mutants (13, 14), which anyway showed a similar left shift for the voltage dependence of activation.

Moreover, the enhancement in the slow inactivation, which prevents prolonged membrane depolarization, is compatible with the observation that none of the patients present episodes of paralysis.

In fact, all probands and affected family members described in this report did not suffer from episodes of paralysis. The clinical presentation includes clinical myotonia and stiffness usually when initiating movement after rest, which worsens with cold exposure in some patients. Myotonia is described as diffuse in the patients with no specific skeletal district, being present in the face, in the hands, and in the lower limbs. Age at onset varies from childhood to adulthood. Muscle strength was normal in all. Some patients carrying p.Ile215Thr showed only electrical myotonia and came to medical attention for an incidental finding. Concomitant paradoxical myotonia may be present in the eyelids as in patient IV.4 from family 1. Only two patients reported weakness: patient II.2 from family 3 complained of mild and transient weakness in whom there was coexisting hypothyroidism so that interpretation of weakness is limited; patient II.1 from family 4 instead complained of progressive and diffuse weakness. Biopsies from Ile215Thr patients were normal or with mild myopathic abnormalities.

The p.Gly241Val patients showed a very different phenotype ranging from the almost asymptomatic mother to her daughter who showed clinical myotonia with muscle pain.

In our cohort of p.Ile215Thr patients, three patients were treated with mexiletine and all of them had good response. Instead, patient II.1 from family 7 that carried p.Gly241Val was treated with mexiletine, acetazolamide, carbamazepine, and phenytoin without success.

The p.Ile215Thr mutation is shared by patients belonging to five out of six unrelated families from Southern Italy. We postulated the existence of a founder effect for this mutation that was demonstrated by marker analysis in the chromosomal region around the *SCN4A* gene. The disease allele harbored by family 2 showed a relatively little region of homology compared to disease allele of families 1 and 3, which could be the result of two different events of chromosomal recombination that independently took place in the past. Another recombination event is shown in patient IV.4 from family 1, indicating that this region is prone to chromosomal recombination. To date, a single case of founder mutation in SCN4A gene has been reported in the French–Canadian population (15).

Recently, homozygous mutations in the *SCN4A* gene were described to cause severe congenital myopathy (12). Before this report, two cases of homozygous patients, one with paramyotonia congenita and one suffering from hypokalemic periodic paralysis, have been reported, leading to severe disability in both patients (16). We describe a new patient affected by SCM with a homozygous mutation in the *SCN4A* gene (patient IV.4, family 1). In our case, homozygosis does not seem to worsen the phenotype compared to heterozygous patients. To explain this counterintuitive phenotypic presentation, beyond an incomplete penetrance of this pathogenic variant that might result in a mild effect when present in a single copy, further modifier elements may be called into question, such as individual genetic background, epigenetic factors (17), and post-translational modifications by miRNA environment (16), each of them able to modulate the effect of the p.Ile215Thr on the phenotype (18). Moreover, the mutations that were described to cause congenital myopathy are loss-of-function mutations that lead to completely non-functional channels due to protein truncation or to the abolished channel function (12).

Given the variability of the myotonic symptoms described by patients carrying mutations in domain I of Na_v_1.4, it is conceivable that the frequency of mutations at this site is underestimated: patients referring for symptoms of locking or stiffness although mild should be screened for SCM. In addition, the finding of a common ancestral disease allele in the Sicilian population is helpful for genetic diagnosis in mild myotonia patients from this region. Antimyotonic treatment may be beneficial and knowledge of a genetic disease will suggest caution when using drugs affecting channel function, i.e., anesthetics, as is recommended for other more severe skeletal channelopathies.

## Data Availability Statement

The raw data supporting the conclusions of this article will be made available by the authors, without undue reservation, to any qualified researcher.

## Ethics Statement

The studies involving human participants were reviewed and approved by Comitato etico Fondazione IRCCS Ca Granda Ospedale Maggiore Policlinico. The patients/participants provided their written informed consent to participate in this study.

## Author Contributions

SPa and GC conceived the study and designed the research. SPa and SL collected the genetic data. ER and MLe collected the electrophysiological data. SPa, SL, ER, MS, MLe, GM, and GC analyzed the data. MS, FM, BF, SPr, VS, GM, AM, and MLo examined the patients. SPa wrote the paper. All authors reviewed and approved the paper.

## Conflict of Interest

The authors declare that the research was conducted in the absence of any commercial or financial relationships that could be construed as a potential conflict of interest. The handling editor declared a past co-authorship with one of the authors with several of the author MS.
